# Return to Soccer After Anterior Cruciate Ligament Reconstruction: An Outcome or a Decision?

**DOI:** 10.1177/23259671241295834

**Published:** 2024-12-18

**Authors:** Isabelle Hållén, Joanna Kvist, Magnus Forssblad, Alexander Sandon

**Affiliations:** * Aleris Sports Medicine and Ortopedi Sabbatsberg, Stockholm, Sweden; † Department of Health, Medicine and Caring Sciences, Division of Physiotherapy, Linköping University, Linköping, Sweden; ‡ Department of Molecular Medicine and Surgery, Stockholm Sports Trauma Research Center, Karolinska Institutet, Stockholm, Sweden; Investigation performed at the Stockholm Sports Trauma Research Center, Department of Molecular Medicine and Surgery, Karolinska Institutet, Stockholm, Sweden

**Keywords:** ACL injury, return to sports, soccer, return to competition, football, ACL surgery

## Abstract

**Background:**

The return-to-sports rate is often used as an outcome measure after anterior cruciate ligament (ACL) reconstruction (ACLR). Although most soccer players want to return to sports after their ACL injury, up to 40% do not believe they will return to soccer after their ACL injury.

**Purpose:**

To investigate whether self-reported presurgical beliefs regarding return to soccer correspond to registered official match participation after ACLR.

**Study Design:**

Cohort study; Level of evidence, 2.

**Methods:**

Included were soccer players with a primary unilateral ACL injury who were ≥15 years at the time of ACLR and who had completed a presurgical question about their future sports participation beliefs. ACLR surgical data were extracted from the Swedish National Knee Ligament Registry, and game participation data were extracted from the Swedish Football Association's administrative data system.

**Results:**

A total of 959 soccer players (617 [64%] male and 342 [36%] female) were included. The follow-up time after ACLR ranged from 18 months to 5.5 years. Of 720 soccer players (75%) who believed that they would return to play (RTP), 462 (64%) players actually did. Of the players who believed that they would not RTP, 181 (76%) did not. Presurgical beliefs predicted RTP (odds ratio [OR], 5.59; *P* < .001). Younger age at the time of ACLR favored RTP, where 61% of the players aged 15-20 years had RTP (OR, 3.85; *P* < .001). At the top competitive level, 84% of the players RTP compared to 14% at the recreational level. Players active at higher levels were more likely to believe that they would RTP, and they also actually did so (OR, 33.06; *P* < .001). Overall, 67% of players followed their presurgical intention to return to soccer.

**Conclusion:**

The findings indicated that presurgical beliefs, age, and level of play helped to predict the actual RTP of soccer players after ACLR.

An anterior cruciate ligament (ACL) injury is a severe sports-related knee injury. Soccer is one of the most common sports in which ACL injuries occur.^38^ Soccer is a popular sport, with more male than female athletes participating; however, the risk of experiencing an ACL injury is 2 to 3 times higher for female players compared to male players. In addition, female athletes also tend to incur the injury at a younger age.^41^

One of the main indications for reconstruction of a torn ACL to restore stability in the knee is the athlete's desire to return to play (RTP) in pivoting sports.^6-8,19,20^ The RTP rate serves as a crucial outcome measure for ACL reconstruction (ACLR) and rehabilitation.^4,6,7,28^ Therefore, exploring factors that can affect the RTP rate is vital.^6,12,17,29^ After ACLR, the majority of athletes report regaining good knee function, but only 50% to 96% actually RTP, with higher numbers for professional athletes. Older age, female sex, cartilage injuries, concomitant or subsequent knee surgery, and pain during physical activity have been presented as negative predictors of RTP.^8,35^ The reasons reported for non-RTP vary. Sandon et al^32^ showed that two-thirds of soccer players who did not RTP reported that it was because of the injured knee, whereas Fältström et al^17^ found that only 14% of female soccer players reported poor knee function as the reason for not returning to play. These authors reported that 28%^32^ and 25%^17^ of the players did not trust their knee because of the fear of a new injury. In most cases, the reason for not returning to play has been discussed as probably being multifactorial^2,17,31,34^ in which psychological factors such as the fear of new injuries and low confidence are important considerations.

High knee-specific self-efficacy^16,21,39^ and great motivation^17,36^ to return to the preinjury level have also been reported as contributing factors for successful RTP. In addition, patients’ expectations have been presented as a prognostic factor for physical recovery^30,36^; at the same time, unrealistically high expectations can lead to postoperative dissatisfaction for the athlete.^10^ As one main reason for undergoing ACLR is to RTP, 84% to 96% of patients expect to RTP.^36,42^ However, results from a 2022 study of patients in the Swedish National Knee Ligament Registry (SNKLR) showed that only 60% of Swedish soccer players reported believing that they would RTP after their ACL injury.^33^

The present study aimed to investigate if self-reported presurgical beliefs toward RTP would correspond to registered official match participation after ACLR, hypothesizing that there would be a significant positive correlation between belief and actual return to participation.

## Methods

### Study Design

After receiving approval from the Swedish Ethical Review Authority for this study's protocol, we identified all patients with an ACL injury who responded to a soccer-specific questionnaire in the SNKLR between April 2017 and August 2020. The criteria for study inclusion were primary unilateral ACLR registered in the SNKLR within 12 months of answering the questionnaire, age ≥15 years at the time of ACLR, registration on a Swedish soccer league team, and possession of a license in the Swedish Football Association's administrative data system, FOGIS. Included patients were also required to have a minimum follow-up of 18 months since ACLR (ie, no surgery date later than March 2021) to ensure at least an 18-month interval between ACLR and RTP.

**Figure 1.**
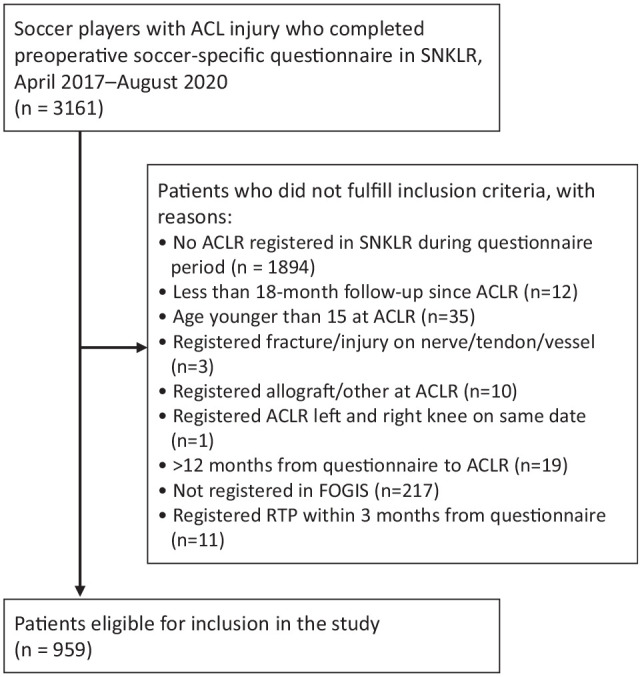
Study flow chart presenting the selection of eligible patients from the SNKLR and FOGIS. SNKLR, Swedish National Knee Ligament Registry; ACL, anterior cruicate ligament; ACLR, ACL reconstruction; RTP, return to play.

A total of 959 soccer players were included (Figure 1). Given the extraction date of registry data in September 2022, the study encompassed follow-up times of up to 5.5 years.

### Data Collection

Registry data from the SNKLR and FOGIS databases were extracted using players’ social security numbers for comparison. Both female and male soccer players meeting the study criteria were included.

#### Swedish National Knee Ligament Registry

Established in 2005, the SNKLR serves as a national surgical registry for ACL injuries, with surgical data entered by orthopaedic surgeons. Currently, 90% of all surgical procedures in Sweden are registered. The SNKLR includes self-reported data and patient-reported outcomes on knee function and quality of life before ACLR and at 1, 2, 5, and 10 years after ACLR. According to the annual report from the SNKLR, the registration rate for presurgical patient-reported outcomes overall is approximately 60%.^38^

The soccer-specific questionnaire, available in the SNKLR since 2017, captures soccer-related details, including beliefs about RTP. The questionnaire was developed in Germany to identify soccer-specific characteristics of ACL injuries after an increase in ACL injuries among German professional male soccer players.^24^ The question “Do you believe that you will continue playing soccer after the ACL injury?” (“yes”/“no” response) measures participants’ RTP beliefs. The participants’ soccer level at the time of the injury, self-registered in the questionnaire, is categorized based on the Swedish soccer league system: “top” (men’s and women's highest and second highest Division), “high” (Division I-II), “middle” (men’s Division III-IV, women's Division III), “low” (women’s Division IV-V, men's Division V-VIII), and “recreational” (recreational sports level).

#### FOGIS Database

The Swedish Football Association's administrative database, FOGIS, is a comprehensive system that contains registered information on player participation in Swedish soccer league games. All players aged ≥15 years must have a registered license in FOGIS to participate in league games. Extracted information from FOGIS included all official soccer games that the study's players participated in and served as an indicator for return to competition after ACLR.

### Statistical Analysis

Statistical analyses were conducted using SPSS Statistics (Version 29; IBM). Descriptive statistics were calculated for all the study variables. Age at the time of ACLR was categorized into 6 groups: 15-20, 21-25, 26-30, 31-35, and ≥36 years. The primary outcome variables (presurgical belief [yes/no] and RTP [yes/no]), both binary in nature, underwent analysis using logistic regression. Adjusted odds ratios (ORs) with 95% confidence intervals were calculated for all covariates in the models. Statistical significance was set at a *P* value of <.05. Sex-specific relationships were explored through separate analyses for female and male players.

## Results

The characteristics of the 959 included soccer players are presented in Table 1. There were 617 (64%) male and 342 (36%) female players, and the follow-up time after ACLR ranged from 18 months to 5.5 years. Overall, 520 (54%) of the players had RTP at a competition level, while 439 (46%) were not. Players who believed before surgery that they would RTP had a significantly higher RTP rate compared to those who did not share this belief (64% vs 24%, respectively; OR, 5.59 [95% CI, 4.01-7.79]; *P* < .001) (Table 1). Of the 720 (75%) players who believed that they would RTP, 462 (64%) successfully returned. The characteristics of these 720 players based on their ability to actually RTP are shown in Table 2.

**Table 1:** Characteristics of Study Population^*a*^

	All Players (n = 959)	Returned to Play		*P*	OR (95% CI)
		Yes (n = 520)	No (n = 439)		
Believed they would RTP				**<.001**	5.59 (4.01-7.79)
Yes^*b*^	720 (75)	462 (64)	258 (36)		
No	239 (25)	58 (24)	181 (76)		
Sex				.833	0.97 (0.75-1.27)
Male^*b*^	617 (64)	333 (54)	284 (46)		
Female	342 (36)	187 (55)	155 (45)		
Age at ACLR, mean ± SD, y	23.0 ± 6.3	22.2 ± 5.7	24.3 ± 6.7	.054	0.98 (0.95-1.00)
Age group at ACLR					
15-20 y^*b*^	391 (41)	237 (61)	154 (39)	**<.001**	3.85 (2.00-7.39)
21-25 y	258 (27)	147 (57)	111 (43)	.356	0.86 (0.63-1.18)
26-30 y	186 (19)	88 (47)	98 (53)	**.003**	0.58 (0.41-0.83)
31-35 y	75 (8)	34 (45)	41 (55)	**.015**	0.54 (0.33-0.89)
≥36 y	49 (5)	14 (29)	35 (71)	**<.001**	0.26 (0.14-0.50)
Associated injuries				.062	0.78 (0.60-1.01)
Yes^*b*^	572 (60)	296 (52)	276 (48)		
No	387 (40)	224 (58)	163 (42)		
Soccer level					
Top^*b*^	58 (6)	49 (84)	9 (16)	**<.001**	33.06 (13.33-81.97)
High	200 (21)	131 (66)	69 (34)	**.007**	0.35 (0.16-0.75)
Middle	253 (26)	151 (60)	102 (40)	**.001**	0.27 (0.13-0.58)
Low	349 (36)	175 (50)	174 (50)	**<.001**	0.18 (0.09-0.39)
Recreational	99 (10)	14 (14)	85 (86)	**<.001**	0.03 (0.01-0.08)

a Data are presented as n (%) unless otherwise indicated. Boldface *P* values indicate a statistically significant difference between RTP groups (*P* < .05) . ACLR, anterior cruciate ligament reconstruction; OR, odds ratio; RTP, return to play.

b Reference category for OR comparisons.

**Table 2:** Characteristics of Players Who Believed That They Would RTP^*a*^

	Believed They Would RTP (n = 720)	Returned to Play		*P*	OR (95% CI)
		Yes (n = 462)	No (n = 258)		
Sex				.544	0.91 (0.66-1.25)
Male^*b*^	464 (64)	294 (63)	170 (37)		
Female	256 (36)	168 (66)	88 (34)		
Age at ACLR, mean ± SD, y	22.4 ± 5.7	22.0 ± 5.4	23.1 ± 6.1	.153	0.98 (0.95-1.01)
Age group at ACLR					
15-20 y^*b*^	320 (44)	213 (67)	107 (33)	.094	2.21 (0.87-5.61)
21-25 y	198 (28)	137 (69)	61 (31)	.535	1.13 (0.77-1.65)
26-30 y	138 (19)	76 (55)	62 (45)	**.020**	0.62 (0.41-0.93)
31-35 y	45 (6)	27 (60)	18 (40)	.386	0.75 (0.40-1.43)
≥36 y	19 (3)	9 (47)	10 (53)	.094	0.45 (0.18-1.15)
Associated injuries				.135	0.79 (0.58-1.08)
Yes^*b*^	420 (58)	260 (62)	160 (38)		
No	300 (42)	202 (67)	98 (33)		
Soccer level					
Top^*b*^	53 (7)	45 (85)	8 (15)	**<.001**	19.22 (7.14-51.70)
High	166 (23)	118 (71)	48 (29)	.049	0.44 (0.19-1.00)
Middle	190 (26)	135 (71)	55 (29)	.046	0.44 (0.19-0.99)
Low	258 (36)	152 (59)	106 (41)	**.001**	0.25 (0.12-0.56)
Recreational	53 (7)	12 (23)	41 (77)	**<.001**	0.05 (0.02-0.14)

a Data are presented as n (%) unless otherwise indicated. Boldface *P* values indicate a statistically significant difference between RTP groups (*P* < .05). ACLR, anterior cruciate ligament reconstruction; OR, odds ratio; RTP, return to play.

b Reference category for OR comparisons.

The remaining 239 (25%) players did not believe that they would RTP after ACLR. Of these, 181 (76%) did not RTP, while 58 (24%) did RTP (Table 3). In total, the expectations about RTP both before and after surgery (either returning or not returning to soccer) aligned for 643 players (67%), while presurgical expectations were not fulfilled for 316 (33%) players.

**Table 3:** Characteristics of Players Who Did Not Believe That They Would RTP^*a*^

	Did Not Believe They Would RTP (n = 239)	Returned to Play		*P*	OR (95% CI)
		Yes (n = 58)	No (n = 181)		
Sex				.557	1.21 (0.65-2.26)
Male^*b*^	153 (64)	39 (25)	114 (75)		
Female	86 (36)	19 (22)	67 (78)		
Age at ACLR, mean ± SD, y	25.5 ± 7.3	24.4 ± 7.3	25.9 ± 7.3	.149	0.96 (0.92-1.01)
Age group at ACLR					
15-20 y^*b*^	71 (30)	24 (34)	47 (66)	.089	2.55 (0.87-7.51)
21-25 y	60 (25)	10 (17)	50 (83)	**.028**	0.39 (0.17-0.91)
26-30 y	48 (20)	12 (25)	36 (75)	.307	0.65 (0.29-1.48)
31-35 y	30 (13)	7 (23)	23 (77)	.300	0.60 (0.22-1.59)
≥36 y	30 (13)	5 (17)	25 (83)	.089	0.39 (0.13-1.15)
Associated injuries				.781	0.92 (0.50-1.69)
Yes^*b*^	152 (64)	36 (24)	116 (76)		
No	87 (36)	22 (25)	65 (75)		
Soccer level					
Top^*b*^	5 (2)	4 (80)	1 (20)	**.001**	88.00 (6.47-1196.20)
High	34 (14)	13 (38)	21 (62)	.112	0.15 (0.20-1.54)
Middle	63 (26)	16 (25)	47 (75)	**.033**	0.09 (0.01-0.82)
Low	91 (38)	23 (25)	68 (75)	**.031**	0.08 (0.01-0.80)
Recreational	46 (19)	2 (4)	44 (96)	**.001**	0.01 (0.00-0.15)

a Data are presented as n (%) unless otherwise indicated. Boldface *P* values indicate a statistically significant difference between RTP groups (*P* < .05). ACLR, anterior cruciate ligament reconstruction; OR, odds ratio; RTP, return to play.

b Reference category for OR comparisons.

### Analysis of Covariates

#### Player Sex

There was no significant difference between female and male players regarding RTP (55% and 54%, respectively) (Table 1) or presurgical beliefs about RTP (75% for both sexes) (Tables 4 and 5).

**Table 4:** Characteristics of Female Players^*a*^

	Female Players (n = 342)	Returned to Play		*P*	OR (95% CI)
		Yes (n = 187)	No (n = 155)		
Believed they would RTP				**<.001**	7.52 (4.16-13.59)
Yes^*b*^	256 (75)	168 (66)	88 (34)		
No	86 (25)	19 (22)	67 (78)		
Age at ACLR, mean ± SD, y	21.1 ± 5.8	20.5 ± 5.3	21.9 ± 6.2	.531	0.99 (0.95-1.03)
Age group at ACLR					
15-20 y^*b*^	199 (58)	118 (59)	81 (41)	.050	3.88 (1.00-15.09)
21-25 y	72 (21)	38 (53)	34 (47)	.338	0.77 (0.45-1.32)
26-30 y	45 (13)	21 (47)	24 (53)	.124	0.60 (0.31-1.15)
31-35 y	15 (4)	7 (47)	8 (53)	.343	0.60 (0.21-1.72)
≥36 y	11 (3)	3 (27)	8 (73)	.050	0.26 (0.07-1.00)
Associated injuries				.071	0.67 (0.44-1.03)
Yes^*b*^	178 (52)	89 (50)	89 (50)		
No	164 (48)	98 (60)	66 (40)		
Soccer level					
Top^*b*^	27 (8)	22 (81)	5 (19)	**<.001**	16.50 (3.80-71.72)
High	137 (40)	90 (66)	47 (34)	.114	0.44 (0.15-1.22)
Middle	67 (20)	38 (57)	29 (43)	**.029**	0.30 (0.10-0.88)
Low	92 (27)	33 (36)	59 (64)	**<.001**	0.13 (0.04-0.37)
Recreational	19 (6)	4 (21)	15 (79)	**<.001**	0.06 (0.01-0.26)

a Data are presented as n (%) unless otherwise indicated. Boldface *P* values indicate a statistically significant difference between RTP groups (*P* < .05). ACLR, anterior cruciate ligament reconstruction; OR, odds ratio; RTP, return to play.

b Reference category for OR comparisons.

**Table 5:** Characteristics of Male Players^*a*^

	Male Players (n = 617)	Returned to Play		*P*	OR (95% CI)
		Yes (n = 333)	No (n = 284)		
Believed they would RTP				**<.001**	5.06 (3.36-7.62)
Yes^*b*^	464 (75)	294 (63)	170 (37)		
No	153 (25)	39 (25)	114 (75)		
Age at ACLR, mean ± SD, y	24.0 ± 6.3	23.2 ± 5.7	25.5 ± 6.7	**.039**	0.97 (0.94-1.00)
Age group at ACLR					
15-20 y^*b*^	192 (31)	119 (62)	73 (38)	**<.001**	4.00 (1.87-8.55)
21-25 y	186 (30)	109 (59)	77 (41)	.502	0.87 (0.57-1.31)
26-30 y	141 (23)	67 (48)	74 (52)	**.009**	0.56 (0.36-0.86)
31-35 y	60 (10)	27 (45)	33 (55)	**.021**	0.50 (0.28-0.90)
≥36 y	38 (6)	11 (29)	27 (71)	**<.001**	0.25 (0.12-0.53)
Associated injuries				.343	0.85 (0.61-1.19)
Yes^*b*^	394 (64)	207 (53)	187 (47)		
No	223 (36)	126 (57)	97 (43)		
Soccer level					
Top^*b*^	31 (5)	27 (87)	4 (13)	**<.001**	47.25 (13.65-163.55)
High	63 (10)	41 (65)	22 (35)	**.031**	0.28 (0.09-0.89)
Middle	186 (30)	113 (61)	73 (39)	**.008**	0.23 (0.08-0.68)
Low	257 (42)	142 (55)	115 (45)	**.002**	0.18 (0.06-0.54)
Recreational	80 (13)	10 (12)	70 (88)	**<.001**	0.02 (0.01-0.07)

a Data are presented as n (%) unless otherwise indicated. Boldface *P* values indicate a statistically significant difference between RTP groups (*P* < .05). ACLR, anterior cruciate ligament reconstruction; OR, odds ratio; RTP, return to play.

b Reference category for OR comparisons.

#### Age at ACLR

The mean age of the players at the time of ACLR was 23.0 ± 6.3 years. There was no significant difference in the RTP rate according to age at the time of ACLR among female players (OR, 0.99 [95% CI, 0.95-1.03]; *P* = .531) (Table 4). However, among male players, younger age at the time of ACLR favored RTP (OR, 0.97 [95% CI, 0.94-1.00]; *P* = .039) (Table 5).

The highest RTP rate occurred among players aged 15-20 years at the time of ACLR, with 237 (61%) of the players had RTP, and lower RTP rates occurred in older age groups (OR, 3.85 [95% CI, 2.00-7.39]; *P* < .001) (Table 1). Among male players, those who were younger had a higher RTP rate than players in older age groups (OR, 4.00 [95% CI, 1.87-8.55]; *P* < .001) (Table 5). No significant difference was observed among female players regarding age.

Soccer players in the youngest age group (15-20 years) were most likely to believe that they would RTP, with 320 (82%) having this belief and 213 (67%) fulfilling this expectation (Table 2). Older age groups were less likely to believe in RTP, although age at the time of ACLR was not found to be significant for presurgical beliefs (Tables 2 and 3).

#### Associated Injuries

Of 959 players, 572 (60%) players had associated injuries, with 296 (52%) of them RTP (Table 1). No significant differences were observed in the RTP rate or presurgical beliefs for RTP between those with an isolated ACL injury and those with associated injuries or among female or male players (Tables 2-5).

#### Soccer Level

Players active at a lower level before the ACL injury were less likely to RTP compared to those active at higher levels before the injury (OR, 33.06 [95% CI, 13.33-81.97]; *P* < .001). At the top level, 49 (84%) of the players returned to play, contrasting with 14 (14%) at the recreational level (Table 1). This trend held true for female and male players separately, with 81% of female players at the top level returning to play compared to 21% at the recreational level (OR, 16.50 [95% CI, 3.80-71.72]; *P* < .001) (Table 4) and 87% of male players at the top level returning to play compared to 12% at the recreational level (OR, 47.25 [95% CI, 13.65-163.55]; *P* < .001) (Table 5).

A significant difference in the soccer level variable was observed regarding presurgical beliefs (OR, 19.22 [95% CI, 7.14-51.70]; *P* < .001) (Table 2). At the top level, 45 (85%) players who believed in RTP actually returned, while the RTP rate decreased at lower soccer levels. At the recreational level, 53 (54%) players thought that they would return, of whom 12 (23%) actually did (Table 2). However, 96% of players in the recreational group who did not believe that they would RTP did not return at a competition level (Table 3).

## Discussion

The principal finding in the present study was that a significantly greater proportion of the soccer players who believed that they would RTP before surgery actually did return compared to those who did not share this belief (64% vs 24%, respectively). In other words, despite only half of the players RTP, 67% of players adhered to their presurgical beliefs. These findings underscore the pivotal role of presurgical beliefs and expectations in influencing postsurgical outcomes.

Overall, 25% of players stated preoperatively that they did not believe that they would RTP. Among these players, 24% did in fact return to competition. While the present study lacks information on the reasons for fulfilling RTP despite presurgical disbeliefs, a reasonable hypothesis is that some players achieve higher motivation^17^ or improved knee-related self-efficacy^21^ during postsurgical rehabilitation. To gain deeper insights into a player's experience and potential contributing factors for fulfilling RTP against initial beliefs, qualitative research would be valuable.

Because of the registry design, we could not provide information about individual reasons for not returning to play among players who initially believed that they would RTP. According to previous studies, the reason for not returning to soccer after ACLR could be knee related. According to Sandon et al,^32^ 1 of 3 athletes did not RTP because of knee problems; other mentioned reasons were change of career, shifting priorities, and family obligations. Reasons for RTP after ACLR have been presented to be multifactorial in other studies.^27,37^ Psychological factors have been reported to have an impact on athletes’ ability to return to sports after ACLR,^18,22,25^ and fear of a new knee injury can be a hindrance for athletes to return to preinjury levels after surgery.^9^ High motivation to RTP has been reported as a positive factor for return to sports after an ACL injury.^5,17,18^

There was no significant difference between male and female players in presurgical beliefs, with 75% of both sexes believing in RTP. This contrasts with previous findings, as Webster and Feller^42^ found that male players had greater presurgical expectations to RTP after ACLR compared to female players (90% vs 84%, respectively). The current study also did not reveal a sex-based difference in the actual RTP rate, which differs from other studies. Ardern et al^7^ reported male athletes to be more likely to return to preinjury levels after ACLR compared to female athletes, and Sandon et al^35^ reported female sex to be a negative predictor for return to soccer after ACLR. In the present study, 36% of the participants were female, which is representative of Swedish ACL injuries. In the annual report from the SNKLR, 34% of the patients experiencing ACL injuries while playing soccer and undergoing primary ACLR were female, and 66% were male.^38^ According to Waldén et al,^40^ female patients experience an ACL injury at a younger age than male patients. Female patients also undergo ACLR at a younger age than male patients.^38^

In the present study, there was no significant sex-based difference in age at the time of ACLR. However, among male players, a younger age at the time of ACLR predicted a higher likelihood of RTP compared to older age at the time of surgery. Soccer players with a younger age at the time of ACLR also exhibited a higher RTP rate than those of older ages. This aligns with previous findings, as Lentz et al^26^ reported older age to be a negative influencing factor regarding RTP after ACLR, and Ardern et al^3^ showed younger age to be favorable for RTP. In most cases, a younger age does not imply the same responsibilities or demands outside of sports participation as for athletes of an older age. At an older age, priorities such as career or family-related liabilities may be a bigger focus,^3^ which may be a reasonable factor for lower RTP rates at higher ages. Previously, Sonesson et al^36^ demonstrated higher motivation during rehabilitation to be associated with RTP.

Associated injuries were common at the time of surgery (60%) but did not significantly affect return to soccer in either female or male players. Having an associated injury in the form of a cartilage injury has earlier been presented to be a negative predictor for RTP after ACLR, without a significant difference in rates of return to soccer related to having a meniscal injury.^35^ Thus, the presence of associated injuries in the knee may delay or influence the RTP rate. Returning to soccer after ACLR implies a higher risk of new injuries,^1,32^ and associated injuries (ie, chondral and meniscal injuries) have been presented as predictive factors for the development of osteoarthritis after ACLR.^13^

Players at higher levels of competition were more likely to RTP, irrespective of their initial beliefs. Those participating at lower levels were less likely to RTP. This result is in line with results from previous studies.^14,15,17,23^ Athletes playing at higher soccer levels often benefit from better access to qualified medical teams, better caretaking, and a shorter time to diagnosis compared to those playing at lower levels, positively influencing the RTP rate.^14^ Being active at higher levels may also include expectations regarding RTP to a greater extent, both from oneself and the social environment, compared to players active at lower levels. The financial incentive and motivators for RTP may also be higher for elite-level players compared to nonelite players.^11^

The findings of this study suggest that, on a group level, a complex interplay of age, level of competition, and individual priorities is a major factor in determining whether the player opts to RTP after ACLR. However, 36% of players did not RTP, despite believing that they would, indicating potential failures related to the knee or a lack of psychological readiness. Understanding the reasons for not returning to play in these athletes requires further investigation beyond the scope of the present study.

In previous studies, one of the main reasons and indicators to recommend an athlete for ACLR, and for the athlete to choose to undergo surgery, has been to enable the player to achieve physical activity demands and return to sports.^19,20^ However, the present study challenges the assumption that all soccer players aspire to RTP after ACLR. The findings revealed that while 75% of players believed that they would RTP, 25% did not believe that they would return. Of these, 24% did actually RTP, although they preoperatively stated that they believed that they would not. This highlights the need to reconsider RTP as the sole objective outcome of the success and effectiveness of ACLR and rehabilitation protocols. The RTP rate was significantly influenced by athletes’ presurgical beliefs toward RTP and the level of competition at which they were engaged, emphasizing the complex interplay between physical recovery and the athlete's mental outlook. Researchers often use the RTP rate as a critical metric for assessing ACLR effectiveness, but the present study challenges this perspective, suggesting that not returning to game participation should not necessarily be seen as a failure.

### Strengths and Limitations

One strength of the present study is the extensive and diverse study population, encompassing both female (n = 342) and male (n = 617) soccer players aged 15 to 47 years, active at various soccer levels. The results of this study can be seen as generalizable to similar pivoting team sports in which an ACL injury is a commonly occurring injury. The study's longitudinal design, combining questionnaire data from the SNKLR and game participation data from FOGIS, contributes to robust findings, as it was possible to control for whether the athlete had returned to playing soccer after ACLR.

Limitations of the present study include the inability to delve into individual reasons for beliefs about RTP because of data constraints and the study design. As the questionnaire is available on the SNKLR website for anyone to voluntarily complete, another limitation is the possible loss of participants in this study because of the low rate of responders. According to the SNKLR's yearly report, 6074 patients who had undergone primary ACLR from 2017 to 2020 sustained their ACL injury while playing soccer, and approximately 50% of these had answered the questionnaire before surgery. While efforts were made to match patients who did not respond to the questionnaire with FOGIS data, the actual player status of nonresponders remained unknown. The follow-up from ACLR ranged from 18 months to 5.5 years. The minimum follow-up was determined to be 18 months to allow for adequate time for RTP, assuming a 12-month rehabilitation period. However, this might result in a loss of participants, as some athletes returned to play after this time frame. Even though some of the registry data were registered during the Coronavirus Disease 2019 pandemic, the study results were not affected by the pandemic.

The study's implications extend to health care professionals, coaches, and sports organizations. Recognizing the pivotal role of presurgical beliefs in RTP decisions can inform tailored rehabilitation strategies and support athletes more effectively. Understanding individual priorities facilitates informed discussions about the potential risks and benefits associated with returning to soccer after ACLR.

## Conclusion

The findings of this study indicated that the presurgical beliefs, age, and level of play of soccer players helped to predict their actual return to game participation after ACLR.

## References

[1] Allen MM Pareek A Krych AJ , et al. Are female soccer players at an increased risk of second anterior cruciate ligament injury compared with their athletic peers? Am J Sports Med. 2016;44(10):2492-2498. 27261476 10.1177/0363546516648439

[2] Ardern CL Österberg A Tagesson S Gauffin H Webster KE Kvist J. The impact of psychological readiness to return to sport and recreational activities after anterior cruciate ligament reconstruction. Br J Sports Med. 2014;48(22):1613-1619. 25293342 10.1136/bjsports-2014-093842

[3] Ardern CL Taylor NF Feller JA Webster KE. Fifty-five per cent return to competitive sport following anterior cruciate ligament reconstruction surgery: an updated systematic review and meta-analysis including aspects of physical functioning and contextual factors. Br J Sports Med. 2014;48(21):1543-1552. 25157180 10.1136/bjsports-2013-093398

[4] Ardern CL Taylor NF Feller JA Webster KE. Return-to-sport outcomes at 2 to 7 years after anterior cruciate ligament reconstruction surgery. Am J Sports Med. 2012;40(1):41-48. 21946441 10.1177/0363546511422999

[5] Ardern CL Taylor NF Feller JA Whitehead TS Webster KE. Sports participation 2 years after anterior cruciate ligament reconstruction in athletes who had not returned to sport at 1 year: a prospective follow-up of physical function and psychological factors in 122 athletes. Am J Sports Med. 2015;43(4):848-856. 25583757 10.1177/0363546514563282

[6] Ardern CL Webster KE Taylor NF Feller JA. Return to sport following anterior cruciate ligament reconstruction surgery: a systematic review and meta-analysis of the state of play. Br J Sports Med. 2011;45(7):596-606. 21398310 10.1136/bjsm.2010.076364

[7] Ardern CL Webster KE Taylor NF Feller JA. Return to the preinjury level of competitive sport after anterior cruciate ligament reconstruction surgery: two-thirds of patients have not returned by 12 months after surgery. Am J Sports Med. 2011;39(3):538-543. 21098818 10.1177/0363546510384798

[8] Balendra G Jones M Borque KA Willinger L Pinheiro VH Williams A. Factors affecting return to play and graft re-rupture after primary ACL reconstruction in professional footballers. Knee Surg Sports Traumatol Arthrosc. 2022;30(7):2200-2208. 34636948 10.1007/s00167-021-06765-8

[9] Cheecharern S. Return to sport and knee functional scores after anterior cruciate ligament reconstruction: 2 to 10 years’ follow-up. Asia Pac J Sports Med Arthrosc Rehabil Technol. 2018;12:22-29. 29963374 10.1016/j.asmart.2018.01.003 PMC6022359

[10] Cole BJ Cotter EJ Wang KC Davey A. Patient understanding, expectations, outcomes, and satisfaction regarding anterior cruciate ligament injuries and surgical management. Arthroscopy. 2017;33(5):1092-1096. 28363420 10.1016/j.arthro.2017.01.049

[11] Creighton DW Shrier I Shultz R Meeuwisse WH Matheson GO. Return-to-play in sport: a decision-based model. Clin J Sport Med. 2010;20(5):379. 20818198 10.1097/JSM.0b013e3181f3c0fe

[12] Czuppon S Racette BA Klein SE Harris-Hayes M. Variables associated with return to sport following anterior cruciate ligament reconstruction: a systematic review. Br J Sports Med. 2014;48(5):356-364. 24124040 10.1136/bjsports-2012-091786 PMC3975129

[13] Diermeier T Rothrauff BB Engebretsen L , et al. Treatment after anterior cruciate ligament injury: Panther Symposium ACL Treatment Consensus Group. Knee Surg Sports Traumatol Arthrosc. 2020;28(8):2390-2402. 32388664 10.1007/s00167-020-06012-6 PMC7524809

[14] Ekstrand J. A 94% return to elite level football after ACL surgery: a proof of possibilities with optimal caretaking or a sign of knee abuse? Knee Surg Sports Traumatol Arthrosc. 2011;19(1):1-2. 21052981 10.1007/s00167-010-1300-4

[15] Erickson BJ Harris JD Cvetanovich GL , et al. Performance and return to sport after anterior cruciate ligament reconstruction in male Major League Soccer players. Orthop J Sports Med. 2013;1(2):2325967113497189. 10.1177/2325967113497189 PMC4555483 26535238

[16] Everhart JS Best TM Flanigan DC. Psychological predictors of anterior cruciate ligament reconstruction outcomes: a systematic review. Knee Surg Sports Traumatol Arthrosc. 2015;23(3):752-762. 24126701 10.1007/s00167-013-2699-1

[17] Fältström A Hägglund M Kvist J. Factors associated with playing football after anterior cruciate ligament reconstruction in female football players. Scand J Med Sci Sports. 2016;26(11):1343-1352. 26589671 10.1111/sms.12588

[18] Gobbi A Francisco R. Factors affecting return to sports after anterior cruciate ligament reconstruction with patellar tendon and hamstring graft: a prospective clinical investigation. Knee Surg Sports Traumatol Arthrosc. 2006;14(10):1021-1028. 16496124 10.1007/s00167-006-0050-9

[19] Grevnerts HT Fältström A Sonesson S Gauffin H Carlfjord S Kvist J. Activity demands and instability are the most important factors for recommending to treat ACL injuries with reconstruction. Knee Surg Sports Traumatol Arthrosc. 2018;26(8):2401-2409. 29411079 10.1007/s00167-018-4846-1 PMC6061764

[20] Grevnerts HT Sonesson S Gauffin H Ardern CL Stålman A Kvist J. Decision making for treatment after ACL injury from an orthopaedic surgeon and patient perspective: results from the NACOX study. Orthop J Sports Med. 2021;9(4):23259671211005090. 10.1177/23259671211005090 PMC8053763 33948447

[21] Hamrin Senorski E Samuelsson K Thomeé C Beischer S Karlsson J Thomeé R . Return to knee-strenuous sport after anterior cruciate ligament reconstruction: a report from a rehabilitation outcome registry of patient characteristics. Knee Surg Sports Traumatol Arthrosc. 2017;25(5):1364-1374. 27530387 10.1007/s00167-016-4280-1 PMC5432591

[22] Heijne A Axelsson K Werner S Biguet G. Rehabilitation and recovery after anterior cruciate ligament reconstruction: patients’ experiences. Scand J Med Sci Sports. 2008;18(3):325-335. 18067526 10.1111/j.1600-0838.2007.00700.x

[23] Krutsch W Lehmann J Jansen P , et al. Prevention of severe knee injuries in men's elite football by implementing specific training modules. Knee Surg Sports Traumatol Arthrosc. 2020;28(2):519-527. 31541292 10.1007/s00167-019-05706-w

[24] Krutsch W Zeman F Zellner J Pfeifer C Nerlich M Angele P. Increase in ACL and PCL injuries after implementation of a new professional football league. Knee Surg Sports Traumatol Arthrosc. 2016;24(7):2271-2279. 25293676 10.1007/s00167-014-3357-y

[25] Kvist J Ek A Sporrstedt K Good L. Fear of re-injury: a hindrance for returning to sports after anterior cruciate ligament reconstruction. Knee Surg Sports Traumatol Arthrosc. 2005;13(5):393-397. 15703963 10.1007/s00167-004-0591-8

[26] Lentz TA Zeppieri G Tillman SM , et al. Return to preinjury sports participation following anterior cruciate ligament reconstruction: contributions of demographic, knee impairment, and self-report measures. J Orthop Sports Phys Ther. 2012;42(11):893-901. 22951437 10.2519/jospt.2012.4077 PMC3680881

[27] Lisee CM DiSanti JS Chan M , et al. Gender differences in psychological responses to recovery after anterior cruciate ligament reconstruction before return to sport. J Athl Train. 2020;55(10):1098-1105. 32966569 10.4085/1062-6050-558.19 PMC7594607

[28] Lynch AD Logerstedt DS Grindem H , et al. Consensus criteria for defining ‘successful outcome’ after ACL injury and reconstruction: a Delaware-Oslo ACL cohort investigation. Br J Sports Med. 2015;49(5):335-342. 23881894 10.1136/bjsports-2013-092299 PMC3900606

[29] Meredith SJ Rauer T Chmielewski TL , et al. Return to sport after anterior cruciate ligament injury: Panther Symposium ACL Injury Return to Sport Consensus Group. Knee Surg Sports Traumatol Arthrosc. 2020;28(8):2403-2414. 32347344 10.1007/s00167-020-06009-1

[30] Mondloch MV Cole DC Frank JW. Does how you do depend on how you think you'll do? A systematic review of the evidence for a relation between patients’ recovery expectations and health outcomes. CMAJ. 2001;165(2):174-179. 11501456 PMC81284

[31] Nwachukwu BU Adjei J Rauck RC , et al. How much do psychological factors affect lack of return to play after anterior cruciate ligament reconstruction? A systematic review. Orthop J Sports Med. 2019;7(5):2325967119845313. 10.1177/2325967119845313 PMC6537068 31205965

[32] Sandon A Engström B Forssblad M. High risk of further anterior cruciate ligament injury in a 10-year follow-up study of anterior cruciate ligament-reconstructed soccer players in the Swedish National Knee Ligament Registry. Arthroscopy. 2020;36(1):189-195. 31439457 10.1016/j.arthro.2019.05.052

[33] Sandon A Krutsch W Alt V Forssblad M. Increased occurrence of ACL injuries for football players in teams changing coach and for players going to a higher division. Knee Surg Sports Traumatol Arthrosc. 2022;30(4):1380-1387. 33987689 10.1007/s00167-021-06604-w PMC9007801

[34] Sandon A Söderström T Stenling A Forssblad M. Can talented youth soccer players who have undergone anterior cruciate ligament reconstruction reach the elite level? Am J Sports Med. 2021;49(2):384-390. 33332148 10.1177/0363546520976651

[35] Sandon A Werner S Forssblad M. Factors associated with returning to football after anterior cruciate ligament reconstruction. Knee Surg Sports Traumatol Arthrosc. 2015;23(9):2514-2521. 24861490 10.1007/s00167-014-3023-4

[36] Sonesson S Kvist J Ardern C Österberg A Silbernagel KG. Psychological factors are important to return to pre-injury sport activity after anterior cruciate ligament reconstruction: expect and motivate to satisfy. Knee Surg Sports Traumatol Arthrosc. 2017;25(5):1375-1384. 27562372 10.1007/s00167-016-4294-8 PMC5432589

[37] Svantesson E Senorski EH Baldari A , et al. Factors associated with additional anterior cruciate ligament reconstruction and register comparison: a systematic review on the Scandinavian knee ligament registers. Br J Sports Med. 2019;53(7):418-425. 30018121 10.1136/bjsports-2017-098192

[38] Swedish National Knee Ligament Registry. Yearly report 2022. Accessed November 7, 2023. Available from: www.aclregister.nu

[39] Thomeé P Währborg P Börjesson M Thomeé R Eriksson BI Karlsson J. Self-efficacy of knee function as a pre-operative predictor of outcome 1 year after anterior cruciate ligament reconstruction. Knee Surg Sports Traumatol Arthrosc. 2008;16(2):118-127. 18034333 10.1007/s00167-007-0433-6

[40] Waldén M Hägglund M Magnusson H Ekstrand J. Anterior cruciate ligament injury in elite football: a prospective three-cohort study. Knee Surg Sports Traumatol Arthrosc. 2011;19(1):11-19. 20532869 10.1007/s00167-010-1170-9

[41] Waldén M Hägglund M Werner J Ekstrand J. The epidemiology of anterior cruciate ligament injury in football (soccer): a review of the literature from a gender-related perspective. Knee Surg Sports Traumatol Arthrosc. 2011;19(1):3-10. 20532868 10.1007/s00167-010-1172-7

[42] Webster KE Feller JA. Expectations for return to preinjury sport before and after anterior cruciate ligament reconstruction. Am J Sports Med. 2019;47(3):578-583. 30649903 10.1177/0363546518819454

